# Assessment of diabetic retinopathy using two ultra-wide-field fundus imaging systems, the Clarus® and Optos™ systems

**DOI:** 10.1186/s12886-018-1011-z

**Published:** 2018-12-20

**Authors:** Takao Hirano, Akira Imai, Hirotsugu Kasamatsu, Shinji Kakihara, Yuichi Toriyama, Toshinori Murata

**Affiliations:** Department of Ophthalmology, Shinshu University School of Medicine, Asahi 3-1-1, Matsumoto, Nagano, 390-8621 Japan

**Keywords:** Diabetic mellitus, Diabetic retinopathy, Ultra-wide-field retinal imaging systems, Early treatment of diabetic retinopathy study diabetic retinopathy severity, International clinical diabetic retinopathy severity

## Abstract

**Background:**

The ability to image wide fundus fields and to conduct swift, non-invasive examinations is increasingly important with the escalation in patients with diabetic retinopathy (DR).

**Methods:**

Fifty eyes of 28 consecutive patients with DR were examined in this prospective observational study. A total of 46 eyes, 25 right and 21 left eyes, of 27 patients (male, 19; female, 8) were ultimately included in the analysis. All patients underwent comprehensive ophthalmological examination. A single image each was obtained using two ultra-wide-field (UWF) imaging systems: Optos® (Optos Carfornia®, Optos PLC, Dunfermline, United Kingdom) and Clarus™ (CLARUS 500™, Carl Zeiss Meditec Inc., Californea, USA), without mydriasis. The total retinal area captured and the obscured retinal area were compared between the two systems using nonparametric Wilcoxon matched-pairs signed-rank analysis. Early Treatment of Diabetic Retinopathy Study (ETDRS) and International Clinical DR severity were analyzed by κ statistics.

**Results:**

The Optos® allowed capture of larger areas of the fundus than the Clarus™ (465 ± 117 vs. 243 ± 39 disc areas*, P* < 0.0001). In 85% (39/46) of Optos® images and 7% (3/46) of Clarus™ images, a slightly obscured area was observed within the ETDRS-7 field area. κ values for ETDRS DR severity and International Clinical DR severity between the Optos® and Clarus™ images were 0.88 and 0.79, respectively. Severity was higher according to Clarus™ images in two eyes in which the ETDRS DR severity grading differed between the systems. Severity was higher in four Clarus™ images and in a single Optos® image in five eyes in which the International Clinical DR severity grading differed between the systems.

**Conclusion:**

The Optos® and Clarus™ UWF retinal imaging systems were useful for examining eyes with DR, using single images obtained without mydriasis. The systems were both generally consistent in assessing DR severity, with some partial discrepancies. It is important to understand the characteristics of each respective UWF retinal imaging system when using them to assess DR.

## Background

Diabetic retinopathy (DR) is the most common cause of vision loss among individuals with diabetes and is the leading cause of vision impairment and blindness among adults of working age [1]. A standard set of definitions that describes DR severity is critical in research settings studies and publications. Early Treatment of Diabetic Retinopathy Study (ETDRS) 7-standard field 35-mm color 30° color fundus images (ETDRS 7-field images) have long been the gold standard for the evaluation of DR severity [2]. However, ETDRS 7-field images require trained photographers, additional time to obtain multiple scans, and good cooperation from the subjects. By contrast, the International Clinical DR severity was proposed as a simplified clinical disease severity scale that can be used internationally [3], and has been used in many reports on DR [4, 5].

Irrespective of which classification is used, it is important to acquire wide-field color fundus images in order to evaluate DR accurately and share information among the relevant parties. Since the number of patients with DR is expected to increase [6], and as early detection and intervention is useful for preventing severe vision loss, it is necessary that the examination can be carried out swiftly and with greater convenience.

According to the Diabetic Retinopathy Clinical Research network, fundus photography with a field that is 100° or more is considered an ultra-wild-field (UWF) [7]. The Optos® (Optos Carfornia®, Optos PLC, Dunfermline, United Kingdom), which is a pioneer in UWF retinal imaging systems, uses a scanning laser ophthalmoscope that can obtain retinal images without mydriasis. This imaging system was designed to cover up to 200° of the retina in a single image. Consequently, many reports have described that the nonmydriatic UWF imaging obtained using the Optos® is useful for evaluation of DR [8–10]. However, some groups have pointed out that eyelash artifacts prevents clear imaging of the inferior periphery [11]. Furthermore, the Optos® fundus image is formed by combination of monochromatic red and green scanning laser ophthalmology scans. Thus, a semi-realistic biocolor Optos® fundus image is sometimes slightly different from a real color image. Since the DR severity changes with the presence or absence of one microaneurysm, these problems might affect the evaluation of DR severity.

The Clarus™ (CLARUS 500™, Carl Zeiss Meditec AG, Jena, Germany) —which is a UWF retinal imaging system designed to cover up to 133° of the retina in a single image—has features of partially confocal optics and true color imaging, which is not available with the Optos®. The partially confocal optics reduces artifacts due to eyelashes and eyelids in retinal images. Additionally, the image formed by the combination of red, green, and blue provides a true color fundus image.

To our knowledge, no previous reports have compared fundus images obtained using the Optos® or Clarus™ UWF retinal imaging systems, based on single images of DR eyes without mydriasis. In this study, these two types of UWF retinal imaging systems were used to examine eyes presenting with DR in terms of the range of the retina captured in the image, the ratio of the area obscured by artifacts, and two measures of DR severity, i.e., the ETDRS DR severity and International Clinical DR severity.

## Methods

This prospective observational study targeted 50 eyes of 28 consecutive patients with DR who visited the outpatient clinic of the ophthalmology department of Shinshu University between October and November 2017. The study was approved by the institutional review board (UMIN 000029098) and adhered to the tenets set forth in the Declaration of Helsinki. Written informed consent was obtained from all patients.

All patients underwent comprehensive ophthalmologic examination, including measurement of best corrected visual acuity (BCVA), slit-lamp biomicroscopy, and color fundus photography using the two UWF imaging systems. Data on age, sex, and previous hemoglobin A1c (HbA1c; National Glycohemoglobin Standardization Program) levels were collected from medical records.

### Retinal image acquisition

UWF fundus imaging was performed in a single-shot without mydriasis and independently of the clinical examination, using the Optos® as well as the Clarus™. All the images were centered on the macula and exported for analysis.

### Evaluation of Total retinal area visualized

**Fig. 1:**
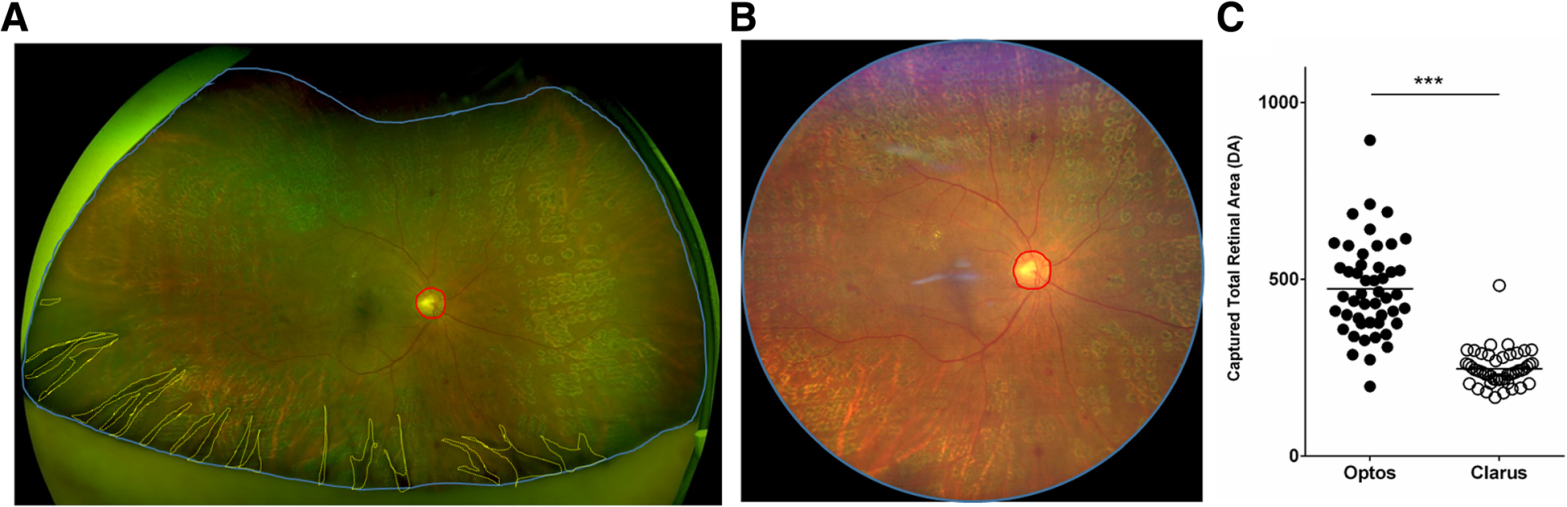
Comparison of captured total retinal area. **a** Retinal image captured by Optos®. **b** Retinal image captured by Clarus™. The blue line excluding the yellow dotted line, which indicates an obscured area, shows the area of visible retina and the red line shows the optic nerve head in (**a**) and (**b**). **c** Captured total retinal area shows significantly higher values in Optos® images than in Clarus™ images (465 ± 117 vs. 243 ± 39 DA, ***P < 0.001). DA: disc area

After importing all the images as a RGB color image (1024 × 1024 pixels) into Image J (National Institutes of Health, Bethesda, MD), images were independently analyzed by two trained readers blinded to patient information (T.H., Y.T.). Using the software, the area of visible retina and optic nerve head were outlined manually and these pixels were quantified. (Fig. 1a, b). The area of visible retina was calculated using the formula: Area of visible retina (disc area [DA]) = Area (pixels) of visible retina/Area (pixels) of optic nerve head**.**

### Evaluation of obscured area

**Fig. 2:**
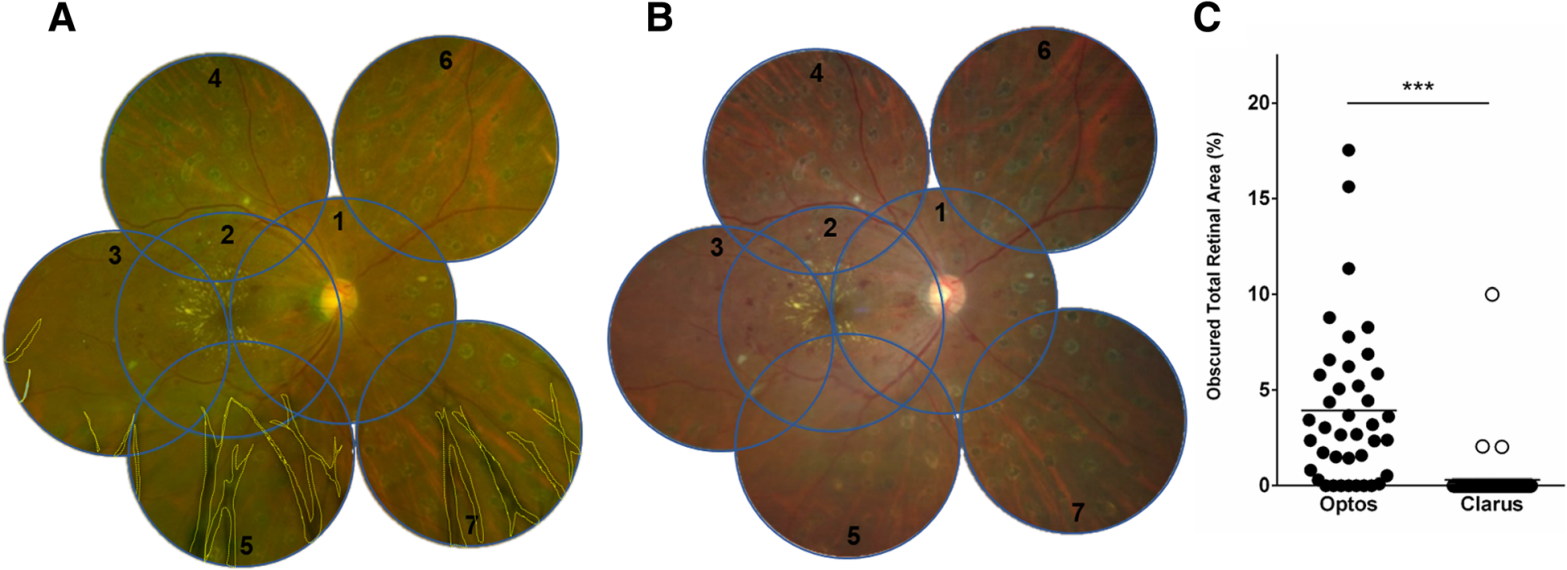
Comparison of obscured retinal area in ETDRS 7-field area. **a** ETDRS 7-field cropped from an Optos® image. **b** ETDRS 7-field cropped from a Clarus™ image. The blue line circles in (**a**) and (**b**) show the ETDRS 7-field. The number in (**a**) and (**b**) indicate the number of the ETDRS 7-field. The yellow dotted lines in (**a**) show the obscured area. **c** Obscured total retinal area was significantly greater in Optos® images than in Clarus™ images (4.15 ± 4.00% vs. 0.47 ± 1.87%, ***P < 0.001). ETDRS: Early Treatment of Diabetic Retinopathy Study

After coverage of ETDRS 7-field images in each UWF images**,** obscured area by artifacts were outlined manually and these pixels were quantified. (Fig. 2 a, b). Obscured area was evaluated using the ratio to the total ETDRS 7-field images area and each field area respectively.

### Grading of images

The ETDRS 7-field area in each of the UWF images were graded for ETDRS DR severity by two independent graders (T.H., Y.T.) according to a previous report [12]. The total area of each of the UWF images were also graded by the same two graders for International Clinical DR severity [3]. Images were shuffled and presented to the graders in random order. The graders were allowed to adjust magnification, brightness, and contrast of the images, but were masked to all additional information. They could decide not to grade an image due to poor image quality, which was defined as an image not covering at least the central 60° and both the macula and optic disc with adequate quality, according to previous reports [10].

Agreement between two graders (T.H., Y.T.) with regard to grading the DR level was demonstrated to be substantial to almost perfect (κ = 0.70 [SE = 0.10] for ETDRS DR severity in Optos images, κ = 0.76 [SE = 0.10] for ETDRS DR severity in Clarus images, and for International Clinical DR severity (κ = 0.72 [SE = 0.10] in Optos images, and κ = 0.84 [SE = 0.08] for Clarus images). A senior retinal specialist (T.M.) independently re-evaluated all images that were scored differently for DR severity by the two graders. After the results were confirmed by the two original graders, the final DR severity was determined and used in analysis.

### Statistical analysis

Statistical analyses were performed using Statistical Packages for Social Sciences 24.0 (IBM, Armonk, NY) and Graph Pad Prism version 6.0 for Windows (Graph Pad Software, San Diego, CA). Continuous variables were expressed as mean values ± standard deviation. Intraclass correlation coefficients (ICCs) were used to estimate the agreement between individual measurements made by the two graders. Since the ICCs between the two readers was consistently > 0.8, comparison of continuous variables between Optos® and Clarus™ images was performed using a nonparametric Wilcoxon matched-pairs signed-rank test, using measurements recorded by one of the readers (Y.T). κ statistics were calculated and assessed based on a previous report: < 0.20, poor; 0.21–0.40, fair; 0.41–0.60, moderate; 0.61–0.80, substantial; and 0.81–1.00, almost perfect agreement [13]. Severity level agreement was cross-tabulated, and κ levels were calculated as previously reported [10]. *P* values < 0.05 were judged to indicate statistical significance.

## Results

### Patient characteristics

Optos® images of four eyes and Clarus™ images of three eyes were not gradable by at least one grader because of poor image quality. A total of 46 eyes, 25 right and 21 left, of 27 patients (male, 19; female, 8) were ultimately included in the analysis. The mean patient age was 61.1 ± 9.6 years (range 39–75 years). In terms of cataract severity, 7 eyes had grade 1 (Emery-Little classification) cataract and 6 eyes had grade 2 cataract; the other 33 eyes had an intraocular lens. Mean visual acuity was 0.25 ± 0.52 log minimum angle of resolution, with a range from − 0.18 to 2.00.

Diabetes duration ranged from 1 to 30, with a mean of 13.4 ± 8.5 years. Fourteen of 27 patients (52%) were using insulin and 44% were on oral medication. Mean HbAc1 was 7.5 ± 2.4% (range 4.4–17.3%). Systemic blood pressure values were 139 ± 22 mmHg (range 105–187 mmHg) systolic and 81 ± 14 mmHg (range 47–115 mmHg) diastolic. In total, 56% (15/27) of the patients were on at least one medication for high blood pressure.

### Evaluation of captured total retinal area

Optos® imaging captured a total retinal area averaging 465 ± 117 DA, with a range of 197 to 713 DA, while the area captured using the Clarus™ imaging was 243 ± 39 DA, and ranged from 166 to 315 DA (*P* < 0.0001) (Fig. 1c).

### Evaluation of obscured retinal area

In 85% (39/46) of Optos® images and 7% (3/46) of Clarus™ images, a slightly obscured area was observed within the ETDRS-7 field area. The obscured total retinal area in the ETDRS-7 field had significantly higher values in Optos® images than in Clarus™ images (4.15 ± 4.00 vs. 0.47 ± 1.87%, *P* < 0.001) (Fig. 2c). A region comprising 6.57 ± 6.17% and 9.52 ± 13.92% of the area in the inferior fundus (fields 5 and 7, respectively) were obscured in the Optos® images (Table 1).

**Table 1:** Existence and proportion of obscured area in each field of the Early Treatment Diabetic Retinopathy Study (ETDRS) 7- field

	Number of ETDRS 7-field							
	1	2	3	4	5	6	7	All field
Optos								
obscured eye *N* (%)	8 (17)	11 (24)	20 (43)	2 (4)	36 (78)	5 (11)	35 (76)	39 (85)
obscured area, %	0.22 ± 0.74	0.41 ± 0.95	1.55 ± 4.18	0.09 ± 0.47	6.57 ± 6.17	1.17 ± 4.75	9.52 ± 13.92	4.15 ± 4.00
Clarus								
obscured eye *N* (%)	0 (0)	0 (0)	0 (0)	2 (4)	1 (2)	3 (7)	2 (4)	3 (7)
obscured area, %	0	0	0	0.13 ± 0.63	0.09 ± 0.64	1.35 ± 7.76	0.04 ± 0.31	0.47 ± 1.87

### Intergrader agreement of retinal area measurements

The interobserver agreement of each retinal area measurement for Optos® and Clarus™ images, as assessed by ICC, was 0.81 (95% confidence interval [CI], 0.68–0.89) and 0.92 (95% CI, 0.85–0.95), respectively; the corresponding ICCs for measurements of the obscured retinal area were 0.88 (95% CI, 0.77–0.94) and 0.97 (95% CI, 0.95–0.98), respectively.

### Comparison of diabetic retinopathy severity levels

The κ value for ETDRS DR severity was 0.88 (SE = 0.08), indicating almost perfect agreement between the Optos® and Clarus™ images. Severity was higher according to Clarus™ images of two eyes of cases where the results of ETDRS DR severity grading differed between the imaging systems (Table 2). The κ value for International Clinical DR severity was 0.79 (SE = 0.09), indicating substantial agreement between the Optos® and Clarus™ images. Severity was higher in four Clarus™ images and in a single Optos® image of five eyes where the results of International Clinical DR severity grading differed between the two imaging systems (Table 3).

**Table 2:** ETDRS DR severity level in ETDRS 7-field images from Optos vs. Clarus

ETDRS DR severity level from Optos	ETDRS DR severity level from Clarus									Total
	10	15/20	35	43	47	53	61	65	71/75	
10	0	0	0	0	0	0	0	0	0	0
15/20	0	0	0	0	0	0	0	0	0	0
35	0	0	**1**	0	0	0	0	0	0	1
43	0	0	0	**1**	0	0	0	0	0	1
47	0	0	0	0	**1**	2	0	0	0	3
53	0	0	0	0	0	**1**	0	0	0	1
61	0	0	0	0	0	0	**37**	0	0	37
65	0	0	0	0	0	0	0	**2**	0	2
71/75	0	0	0	0	0	0	0	0	**1**	1
Total	0	0	1	1	1	3	37	2	1	46

Boldface numbers indicate perfect agreement

*ETDRS* Early treatment of diabetic retinopathy study, *DR* diabetic retinopathy

**Table 3:** International clinical DR severity level in whole images acquired from Optos vs. Clarus

International clinical DR severity level from Optos	International clinical DR severity level from Clarus					Total
	No DR	Mild NPDR	Moderate NPDR	Severe NPDR	PDR	
No DR	0	0	0	0	0	0
Mild NPDR	0	0	1	0	0	1
Moderate NPDR	0	0	**27**	3	0	30
Severe NPDR	0	0	1	**10**	0	11
PDR	0	0	0	0	**4**	4
Total	0	0	29	13	4	46

Boldface numbers indicate perfect agreement

*DR* diabetic retinopathy, *NPDR* nonproliferative diabetic retinopathy, *PDR* proliferative diabetic retinopathy

## Discussion

In this study, two types of UWF retinal imaging systems, the Optos® and Clarus™ systems, were used to examine DR eyes, using single images obtained without mydriasis. In all, 92% (46/50) of images captured using the Optos® system and 94% (47/50) of images obtained using the Clarus™ system were suitable for use in assessing DR severity. The Optos® system allowed for capture of larger areas of the fundus than the Clarus™ system, which was plausible, as the maximum capture range for the Optos® system was 200°, but only 133° for the Clarus™ system. In ETDRS 7-field images, a greater area was obscured by artifacts in the Optos® images than in the Clarus™ images. It is likely that the partially confocal optics function included in the Clarus™ system will be capable of reducing lid and lash artifacts. The agreement in ETDRS DR severity between the Optos® and Clarus™ images was almost perfect. Interestingly, the DR severity determined from Clarus™ images was higher than that determined from Optos® images in two cases. In these cases, some portions of the retina could not be examined in the Optos® images due to the presence of artifacts, and the cause of this discrepancy was determined to be retinal hemorrhaging, which could be observed using the Clarus™ images, but which was obscured in the Optos® images.

The agreement between the two systems was also substantial in terms of the International Clinical DR severity. Severity was greater in four Clarus™ images and in a single Optos® image of five eyes where the results of the International Clinical DR severity grading differed between the systems. As we had anticipated that severity would be greater in Optos® images for International Clinical DR severity grading because the Optos® system can capture wider images than the Clarus™ system, this was an unexpected result.

Considering the higher DR severity observed in the Clarus™ images, we believe that the cause of the partial obscuration by artifacts in the Optos® images, which was similar to that affecting ETDRS DR severity (mainly in the inferior regions, as previously reported [14]), was microaneurysm/retinal hemorrhage that could be detected only in Clarus™ images. In contrast, a large volume of peripheral retinal hemorrhage was confirmed in the single case where the DR severity was found to be greater according to the Optos® image. As described previously, the effect of artifacts can be mitigated using the partially confocal optics functionality and instances of microaneurysm/retinal hemorrhage can be depicted more clearly using true color imaging, which is possible with the Clarus™. As such, when conducting assessments requiring limited-field single images obtained without mydriasis, such as when assessing ETDRS DR severity, the Clarus™ system may be more suitable than the Optos® system. However, recent reports have suggested that the Optos® system can identify additional retinal abnormalities in patients with DR [15]. In this study, peripheral retinal hemorrhage that could not be detected by the Clarus™ system could be identified using the Optos® system, as evidenced by the single case in which the International Clinical DR severity was increased. Additionally, the presence of peripheral lesions in DR eyes is associated with an increased risk for DR progression [16]. Thus, the Optos® system may be more suitable than the Clarus™ system for assessments requiring wider retinal imaging ranges, such as when assessing the International Clinical DR severity.

This study had several limitations. The sample size was relatively small. Additionally, as our study site was a university hospital, there were many patients with very severe DR. For patients with low DR severity, the severity could vary by one microaneurysm/retinal hemorrhaging; this may have produced more notable differences between the two imaging systems. Thus, examining a cohort with varying degrees of severity will be necessary in future studies.

We did not demonstrate the full potential of each system. As we attempted to obtain fundus images rapidly and non-invasively from DR patients, we acquired only single images without mydriasis. Mydriatic images taken using the Optos® system are reportedly of better quality than non-mydriatic images [9]. Additionally, combining Optos® images of different parts of the retina allows capture of a wider retinal field, with fewer artifacts [14]. The Clarus™ system can combine images of different regions of the retina.

Additionally, the separated color scan of the Optos® image potentially provides additional information, as the green, “red-free” scan may include more selective information about the superficial layers of the neurosensory retina [17, 18]. Although the Optos® images were less useful than the Clarus™ images for identifying microaneurysm/retinal hemorrhage, it was possible to improve the utility of these images via the separated color scan images.

For single images obtained without mydriasis to examine eyes with DR, the Optos® and Clarus™ UWF retinal imaging systems are both useful. The Optos® allowed imaging of a wider fundus field than the Clarus™. The Clarus™ produced fewer artifacts and yielded more detailed imaging of the fundus than the Optos®. While both systems demonstrated generally favorable consistency in assessments of DR severity, some partial discrepancies were noted. It is therefore important to understand the characteristics of each respective UWF fundus imaging system when using them to assess DR.

## Conclusion

The Optos® and Clarus™ UWF retinal imaging systems were useful for examining eyes with DR, using single images obtained without mydriasis. The systems were both generally consistent in assessing DR severity, with some partial discrepancies. It is important to understand the characteristics of each respective UWF retinal imaging system when using them to assess DR.

## Abbreviations

BCVA: Best corrected visual acuity
DA: Disc area
DR: Diabetic retinopathy
ETDRS: Early Treatment of Diabetic Retinopathy Study
HbA1c: Hemoglobin A1c
ICCs: Intraclass correlation coefficients
NPDR: Nonproliferative diabetic retinopathy
PDR: Proliferative diabetic retinopathy
UWF: Ultra-wide-field
